# Epstein-Barr virus latent membrane protein 2A suppresses the expression of HER2 via a pathway involving TWIST and YB-1 in Epstein-Barr virus-associated gastric carcinomas

**DOI:** 10.18632/oncotarget.2702

**Published:** 2014-11-06

**Authors:** Yi-wang Zhang, Xiao-xiao Zhao, Cui Tan, Zhi-gang Zhang, Ye Jiang, Jian-ning Chen, Hong-bo Wei, Ling Xue, Hai-gang Li, Hong Du, Chun-kui Shao

**Affiliations:** ^1^ Department of Pathology, The Third Affiliated Hospital, Sun Yat-sen University, Guangzhou, Guangdong Province, China; ^2^ Department of Gastrointestinal Surgery, The Third Affiliated Hospital, Sun Yat-sen University, Guangzhou, Guangdong Province, China; ^3^ Department of Pathology, The First Affiliated Hospital, Sun Yat-sen University, Zhongshan Guangzhou, Guangdong Province, China; ^4^ Department of Pathology, Sun Yat-sen Memorial Hospital, Sun Yat-sen University, Guangzhou, Guangdong Province, China; ^5^ Department of Pathology, Guangzhou First Municipal People's Hospital, Guangzhou, Guangdong Province, China

**Keywords:** EBVaGC, HER2, LMP2A, TWIST, YB-1

## Abstract

To explore HER2 expression in Epstein-Barr virus-associated gastric carcinoma (EBVaGC) and the possible mechanisms causing down-regulation of HER2 expression in EBVaGC, we first evaluated HER2 and LMP2A expression on a clinicopathological-features matched cohort including 78 EBVaGC and 216 EBV-negative gastric carcinoma (EBVnGC) cases by immunohistochemistry. Cases with high HER2 expression in EBVaGC were significantly less than in EBVnGC (5.1% versus 23.7%; p<0.001), and none of the 34 LMP2A+ EBVaGC showed high HER2 expression. Further, overexpressing LMP2A in EBV-negative SGC7901 cells significantly decreased HER2, TWIST and YB-1 mRNA by 36.1%±8.1%, 87.6%±14.0% and 83.8%±5.7%, and protein by 44%, 57% and 49%, respectively. Additionally, the nucleus/cytoplasm ratios of TWIST and YB-1 were also decreased by 85% and 80%, respectively. Silencing LMP2A by siRNA in EBV-positive SNU719 cells for 48 h significantly increased HER2, TWIST and YB-1 mRNA to 276.7%±14.6%, 1284.8%±38.2% and 332.0%±15.5% and protein to 212%, 457% and 232%, respectively. The nucleus/cytoplasm ratios of TWIST and YB-1 were up-regulated by 4.00- and 3.57-fold, respectively, following LMP2A down-regulation. Moreover, LMP2A+/HER2^low^ EBVaGC cases presented the best overall survival compared with LMP2A−/HER2^low^ and LMP2A−/HER2^high^ cases (p=0.003, log-rank test). These results suggest that LMP2A may suppress the HER2 expression through the TWIST/YB-1 axis in EBVaGC.

## INTRODUCTION

EBV is a ubiquitous human herpes virus that causes a life-long asymptomatic persistent infection in more than 95% of the human population [1]. EBV is thought to be closely associated with the development of some lymphoid neoplasms, nasopharyngeal carcinoma (NPC) and EBVaGC [2,3]. EBVaGC is defined as the presence of EBV in gastric carcinoma cells by EBV-encoded RNA (EBER) *in situ* hybridization [4,5]. EBVaGC comprise approximately 10% of all gastric carcinomas worldwide with a frequency ranging from 1.3% to 20.1% in different countries [6,7]. Our previous study suggested that the proportion of EBVaGC in gastric carcinoma in Guangzhou, southern China, where NPC is endemic, was 6.7% (45/676) [8]. EBVaGC has several distinct clinicopathological features, such as being highly associated with the male gender and younger individuals, presenting generally diffuse-type histology and frequently occurring in the gastric cardia and body [5,9]. Previous studies have not reached a consensus on the prognosis for EBVaGC. Camargo MC *et al.* have reported that EBVaGC exhibited a significantly longer survival time compared with EBVnGC [10,11], while van Beek *et al.* and Huang SC *et al.* observed no difference in overall survival between EBVaGC and EBVnGC [12,13].

EBV persists in hosts in a latency cycle and constitutively expresses a limited set of viral gene products, the so-called latent products, which are composed of six EBV nuclear antigens (EBNAs 1, 2, 3A, 3B, 3C and EBNA-LP), three latent membrane proteins (LMPs 1, 2A and 2B), two EBV-encoded small non-coding RNAs (EBERs 1 and 2) and the BamHI A rightward transcripts (BARTs). Depending on the expression of different combinations of the latent products, three latency patterns have been classified [2,4]. Latency І is limited to only EBERs, BARTs and EBNA1 expression; latency II includes LMP1 and LMP2 in addition to the latency І expressed products; and latency III is defined by the expression of all of the latent products [2]. EBVaGC is a latency I neoplasm and expresses EBNA1, EBERs and BARTs [4,14]. In addition, nearly half of EBVaGC cases express LMP2A [4].

LMP2A, which is encoded by the LMP2 gene, consists of 497 amino acids (aa), and interacts with cellular proteins mainly through its long N-terminal tail [4,14,15]. LMP2A may contribute to up-regulation of oncogenes, such as the Ras gene, or down-regulation of tumor suppressor gene, such as PTEN [16,17].

Human epidermal growth factor receptor-2 (HER2), a known proto-oncogene located on the long arm of human chromosome 17 (17q12), is a member of the epidermal growth factor receptor (EGFR) superfamily associated with tumor cell proliferation, apoptosis, adhesion, migration, and differentiation [18,19]. HER2 is overexpressed in many human epithelial malignancies, including breast cancer, ovarian cancer, bladder cancer and gastric carcinoma [20,21]. In gastric carcinomas, HER2 overexpression was associated with poor outcomes and aggressive disease [18,22]. In 2010, an open-label, phase 3, randomized controlled trial of trastuzumab in combination with chemotherapy versus chemotherapy alone for treatment of HER2-positive advanced gastric or gastro-esophageal junction cancer (ToGA) was undertaken in 122 centers in 24 countries. This ToGA study determined that 22.1% (810/3665) of gastric cancer cases overexpressed HER2, and trastuzumab, a monoclonal antibody that targets HER2, could be considered a new standard option for patients with HER2-positive gastric carcinomas and be helpful for improving survival in these patients [23].

Due to the pivotal role that HER2 plays in tumor growth and its relationship with therapeutic efficacy in gastric carcinomas, and because EBVaGC was recently proposed as a subtype of gastric carcinoma [24], we wanted to examine the expression of HER2 in EBVaGC. Thus far, only a few studies have focused on this issue. Lee HS *et al.* from Korea showed that 1/63 (1.6%) EBVaGC cases exhibited HER2 overexpression, and 38/281 (13.5%) EBVnGC cases showed HER2 overexpression [25]. Sukawa Y *et al.* in Japan revealed that HER2 overexpression was found in one (5.6%) out of 18 EBVaGC cases and 19 (8.9%) out of 213 EBVnGC cases [26]. However, the clinicopathological features of EBVaGC and EBVnGC cases in both studies were not matched, and these unmatched clinicopathological features were not beneficial to reveal the influence of EBV on HER2 expression in the univariate analysis.

In this study, we explored HER2 expression in 78 EBVaGC and 216 EBVnGC clinicopathologically-matched cases by immunohistochemistry. We found a significantly reduced expression of HER2 in EBVaGC. Further, this reduction in HER2 expression may be induced by LMP2A, one of the EBV latency proteins, in EBVaGC through a pathway involving TWIST and YB-1. Moreover, LMP2A+/HER2^low^ EBVaGC cases presented the best overall survival compared with LMP2A−/HER2^low^ and LMP2A−/HER2^high^ EBVaGC cases.

## RESULTS

### Suppression of HER2 expression in EBVaGC cases

EBVnGC cases were selected so that their clinicopathological features (sex, age, tumor differentiation, and TNM classification) statistically matched the EBVaGC cases (Table 1). Additionally, this matching of clinicopathological features ensured that further analysis of EBV effecting HER2 expression was univariate.

**Table 1:** Clinicpathological characteristics and HER2 expression in EBVaGC and EBVnGC

Characteristic	EBVaGC N (%)	EBVnGC N (%)	^†^*p*
Gender			0.498
Male	57 (84.6)	149 (69.0)	
Female	21 (15.49)	67 (31.0)	
Age (years)			0.054
≤40	17 (21.8)	26 (12.0)	
40~60	42 (53.8)	114 (52.8)	
>60	19 (24.4)	76 (35.2)	
Mean ± SD^‡^	52.4±13.6	55.1±12.0	
Lauren			0.079
Intestinal	18 (23.1)	73 (33.8)	
Diffuse	60 (76.9)	143 (66.2)	
Clinical Stage (pTNM)^||^			0.142
I, II	20 (25.6)	75 (34.7)	
III, IV	58 (74.4)	141 (65.3)	
Invasion			
T1,T2	10 (12.9)	46 (21.3)	0.102
T3,T4	68 (87.1)	170 (78.7)	
Nodal status			
N−	20 (25.6)	64 (29.6)	0.504
N+	58 (74.4)	152 (70.4)	
Distant metastasis			
M0	76 (97.4)	199 (92.1)	0.106
M1	2 (2.6)	17 (7.9)	
HER2			<0.001
0/1+	74 (94.9)	165 (76.3)	
2+	3 (3.8)	31 (14.4)	
3+	1 (1.3)	20 (9.3)	

† *p*-values were obtained from Pearson Chi-Square tests, Fisher's exact tests or Kruskal Wallis Test.

‡ SD: standard deviation.

|| AJCC TNM cancer staging system.

**Figure 1:**
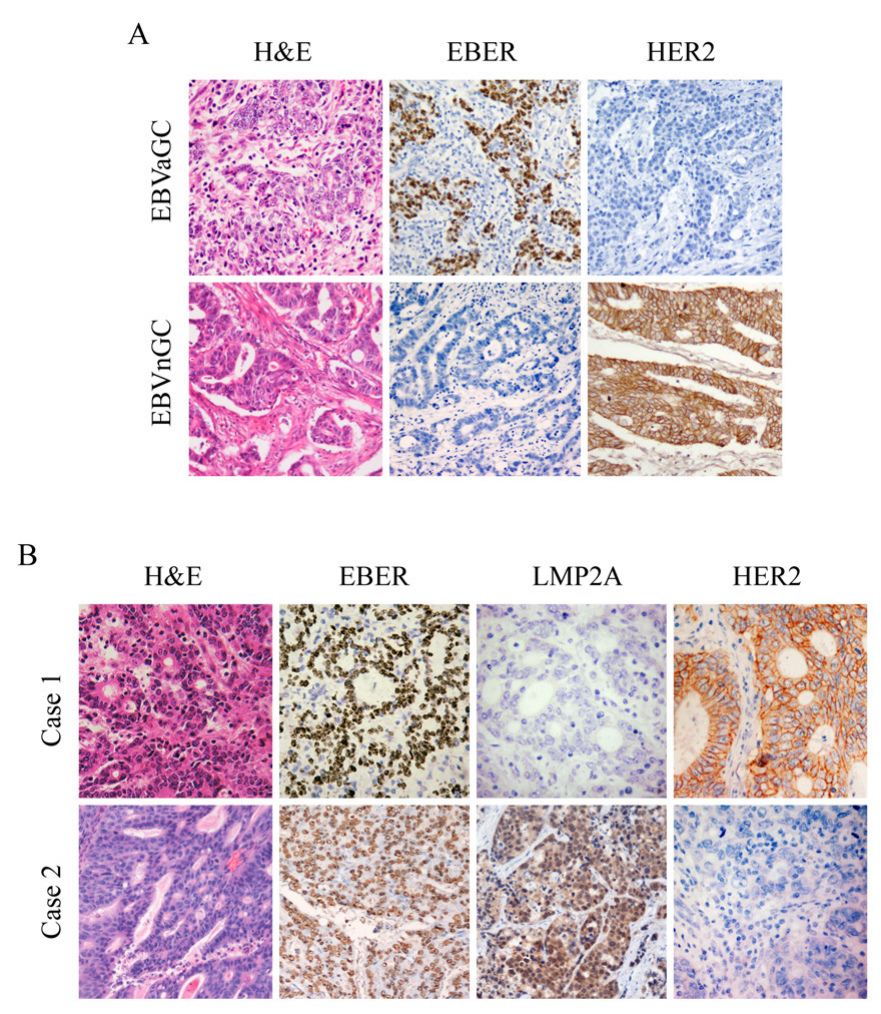
IHC images for expression of HER2, EBER and LMP2A in gastric tumor specimens Each of the gastric carcinoma cases was stained by H&E, IHC and EBER-1 *in situ* hybridization, respectively. For EBER-1 *in situ* hybridization, the positive signals were restricted only to the tumor nuclei but not in surrounding non-tumor cells. For HER2 IHC staining, positive staining can be seen in the membrane of the tumor cells. (*A*) Representative images for expression of HER2 in EBVaGC and EBVnGC samples. (*B*) Representative images of LMP2A and HER2 expression in EBVaGC samples. Case 1 showed negative expression of LMP2A with overexpression of HER2 (3+), and case 2 exhibited positive expression of LMP2A with no apparent expression of HER2 (0). *(Original magnification ×200).*

Gastric carcinoma samples were defined as high (2+, 3+) or low (0, 1+) expression of HER2 based on IHC results [23,27]. EBVaGC cases exhibited significantly less expression of HER2 than EBVnGC cases (5.1% versus 23.7%; p<0.001; Table 1). Representative IHC images of both EBER and HER2 expression in cancer cells are presented in Figure 1A.

Furthermore, clinicopathological characteristics of the EBVaGC and EBVnGC cases were evaluated based on HER2 expression. There were no significant differences observed in sex, age, tumor differentiation, or TNM classification between the HER2 2/3+ group and HER2 0/1+ group in EBVaGC (Table 2). However, cases with high HER2 expression presented significantly more distant metastases than low HER2 expression in EBVnGC (p=0.038; Table 2).

**Table 2:** Clinicopathological characteristics of patients with EBVaGC or EBVnGC based on HER2 expression

	EBVaGC		^†^*P*	EBVnGC		^†^*P*
	HER2 0/1+ n=74	HER2 2/3+ n=4		HER2 0/1+ n=165	HER2 2/3+ n=51	
Gender			1.000			0.626
Male	53 (71.6)	4 (100)		106 (64.2)	33 (64.7)	
Female	12 (28.4)	0		49 (35.8)	18 (35.3)	
Age (years)			0.805			0.664
≤40	17 (23.0)	0		20 (12.1)	6 (11.8)	
40~60	39 (52.7)	3 (75.0)		82 (49.7)	22 (43.1)	
>60	18 (24.3)	1 (25.0)		63 (38.2)	23 (45.1)	
Lauren			0.226			0.349
Intestinal	16 (21.6)	2 (50.0)		53 (32.1)	20 (39.2)	
Diffuse	58 (78.4)	2 (50.0)		112 (67.9)	31 (60.8)	
Clinical Stage (pTNM)^||^			1.000			0.565
I, II	19 (25.7)	1 (25.0)		59 (35.8)	16 (31.4)	
III, IV	55 (74.3)	3 (75.0)		106 (64.2)	35 (68.6)	
Invasion			1.000			0.263
T1,T2	10 (13.5)	0		38 (23.0)	8 (15.7)	
T3,T4	64 (86.5)	4 (100)		127 (77.0)	43 (84.3)	
Nodal status			1.000			0.275
N−	14 (18.9)	1 (25.0)		52 (31.5)	12 (23.5)	
N+	60 (81.1)	3 (75.0)		113 (68.5)	39 (76.5)	
Distant metastasis			1.000			0.038
M0	72 (97.3)	4 (100)		156 (94.5)	43 (84.3)	
M1	2 (2.7)	0		9 (5.5)	8 (15.7)	

† *p*-values were obtained from Pearson Chi-Square tests, Continuity Correction or Fisher's exact tests.

|| AJCC TNM cancer staging system.

### Clinicopathological features and expression of HER2 in EBVaGC cases based on LMP2A

LMP2A was localized on the cell surface and in the cytoplasm of tumor cells (Figure 1B). Within the 78 EBVaGC, there were 34 cases that exhibited positive LMP2A expression (43.6%) by IHC. No significant correlations in sex, age, tumor differentiation, or TNM classification were found between LMP2A-positive and LMP2A-negative cases (Table 3). Further, none of the 34 LMP2A-positive EBVaGC cases showed high-expression of HER2 and 4 of 44 LMP2A-negative EBVaGC cases (9.1%) exhibited high-expression of HER2. The rate of High HER2 expression in LMP2A-positive EBVaGC cases was not significantly different from that in LMP2A-negative EBVaGC cases (p=0.073; Table 3).

**Table 3:** Clinicopathological characteristics of patients with EBVaGC based on LMP2A expression

	LMP2A Positive N (%)	LMP2A Negative N (%)	^†^*p*
Gender			0.08
Male	26 (76.5)	40 (90.9)	
Female	8 (23.5)	4 (9.1)	
Age (years)			0.465
≤40	6 (17.5)	11 (25.0)	
40~60	21 (61.9)	21 (47.7)	
>60	7 (20.6)	12 (27.3)	
Lauren			0.934
Intestinal	8 (23.5)	10 (22.7)	
Diffuse	26 (76.5)	34 (77.3)	
Clinical Stage (pTNM)^||^			0.503
I, II	10 (29.4)	10 (22.7)	
III, IV	24 (70.6)	34 (77.3)	
Invasion			0.436
T1,T2	6 (17.6)	4 (9.1)	
T3,T4	28 (82.4)	40 (90.9)	
Nodal status			1.000
N−	7 (20.6)	9 (20.5)	
N+	27 (79.4)	35 (79.5)	
Distant metastasis			1.000
M0	34 (100)	42 (95.5)	
M1	0	2 (4.5)	
HER2			0.073
0/1+	34 (100)	40 (90.9)	
2+	0	3 (6.8)	
3+	0	1 (2.3)	

† *p*-values were obtained from Pearson Chi-Square tests, Continuity Correction or Kruskal Wallis Test.

|| AJCC TNM cancer staging system.

Detailed clinicopathological features of the 4 HER2 high-expressing EBVaGC cases are displayed in Table 4.

**Table 4:** Clinicopathological characteristics of 4 EBVaGC cases with HER2 high-expression

Case	Gender	Age(years)	Lauren	Clinical Stage (pTNM)	Invasion	Nodal status	Distant metastasis	HER2
1	M	45	dif	IIIA	T3	N2	M0	2+
2	M	57	dif	IIIA	T3	N2	M0	2+
3	M	58	int	IIIB	T3	N3	M0	3+
4	M	76	int	IIA	T3	N0	M0	2+

Abbreviations: F, female; M, male; dif, Diffuse type; int, Intestinal type.

### Overexpression of LMP2A down-regulates HER2, TWIST and YB-1 expression in GC cells

**Figure 2:**
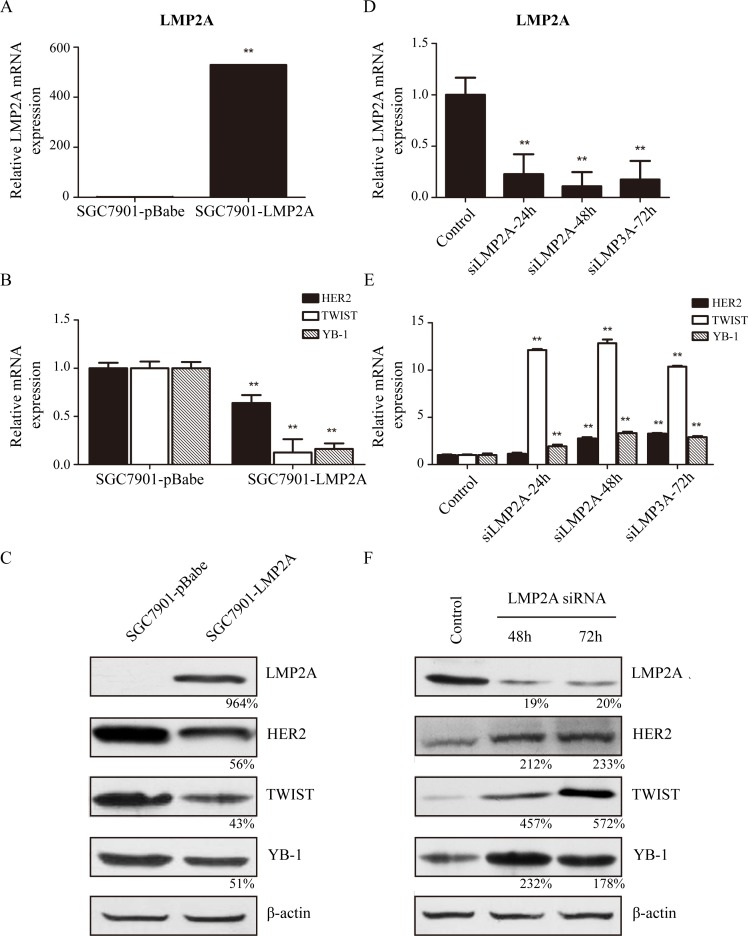
LMP2A inhibited expression of HER2, TWIST and YB-1 on both transcriptional and translational levels SGC7901 cells were stably nucleofected with an empty pBabe vector or vector subcloned with LMP2A. (*A*) qRT-PCR suggested that level of LMP2A mRNA was up-regulated in five hundred fold in SGC7901-LMP2A cells (^**^p<0.001 vs SGC7901-pBabe cells). (*B*) Following LMP2A mRNA levels increased, the mRNA levels of HER2, TWIST and YB-1 were significantly decreased (^**^p<0.001 vs SGC7901-pBabe cells). (*C*) Western blot analysis shows that the protein levels of HER2, TWIST and YB-1 were also reduced after LMP2A was exogenous overexpressed in SGC7901-LMP2A cells relative to SGC7901-pBabe cells. SNU719 cells were transiently nucleofected with LMP2A siRNA or a universal siRNA control for 24, 48 and 72 h. (*D*) qRT-PCR showed that the mRNA level of LMP2A in SNU719 siLMP2A cells was down-regulated after interfering 24h, and reduced by 89.1% at 48h (^**^p<0.001 vs control SNU719 cells). (*E*) When LMP2A mRNA levels decreased, mRNA levels of TWIST and YB-1 were significantly up-regulated by 12.84- and 3.32-fold at 48h, and HER2 mRNA increased by 3.28-fold at 72h (^**^p<0.001 vs control SNU719 cells). (*F*) Western blot analysis exhibited that the protein levels of HER2, TWIST and YB-1 were also increased after LMP2A was siRNA silenced in SNU719 siLMP2A cells relative to control SNU719 cells.

Subsequently, SGC7901 cells were stably transfected with the LMP2A gene as described in the materials and methods. SGC7901-LMP2A cells exhibited a significant and reproducible up-regulation of LMP2A mRNA levels (528.9%±2.3%; Figure 2A) relative to SGC7901-pBabe cells, and the transcriptional levels of HER2, TWIST and YB-1 were down-regulated by 36.1%±8.1%, 87.6%±14.0% and 83.8%±5.7%, respectively, in SGC7901-LMP2A cells (Figure 2B). Western blotting analysis showed that HER2, TWIST and YB-1 decreased by 44%, 57% and 49%, respectively, in SGC7901-LMP2A cells following LMP2A up-regulation (Figure 2C). These results suggested that the exogenous overexpression of LMP2A in SGC7901 cells could suppress the expression of HER2, TWIST and YB-1 at the transcriptional level.

### LMP2A-targeted siRNA up-regulated HER2, TWIST and YB-1 expression in GC cells

Next, SNU719 cells were transiently nucleofected with LMP2A siRNA or a universal siRNA control. qRT-PCR tests showed that LMP2A mRNA levels were decreased by 77.2%±19.3%, 89.1%±13.9% and 82.4%±17.9% at 24, 48 and 72 h, respectively, in siLMP2A-transfected SNU719 cells compared with universal siRNA control-transfected SNU719 cells (Figure 2D). Meanwhile, at 24, 48 and 72 h in siLMP2A-transfected SNU719 cells, the mRNA level of HER2 was increased to 114.0%±11.5%, 276.7%±14.6% and 328.2%±6.1%, respectively, the mRNA level of TWIST was up-regulated to 1211.3%±11.2%, 1284.8%±38.2% and 1037.2%±8.6%, respectively, and the YB-1 mRNA level was increased to 193.5%±16.8%, 332.0%±15.5% and 289.0%±12.0%, respectively (Figure 2E). Further, Western blotting analysis showed that LMP2A expression was decreased by 81% at 48 h in siLMP2A-transfected SNU719 cells, and HER2, TWIST and YB-1 expression was increased to 212%, 457% and 232%, respectively (Figure 2F).

These results demonstrate that silencing of LMP2A by siRNA in SNU-719 cells may induce the up-regulation of HER2, TWIST and YB-1 expression at the transcriptional level.

### Influence of LMP2A overexpression or silencing on TWIST and YB-1 intracellular localization

**Figure 3:**
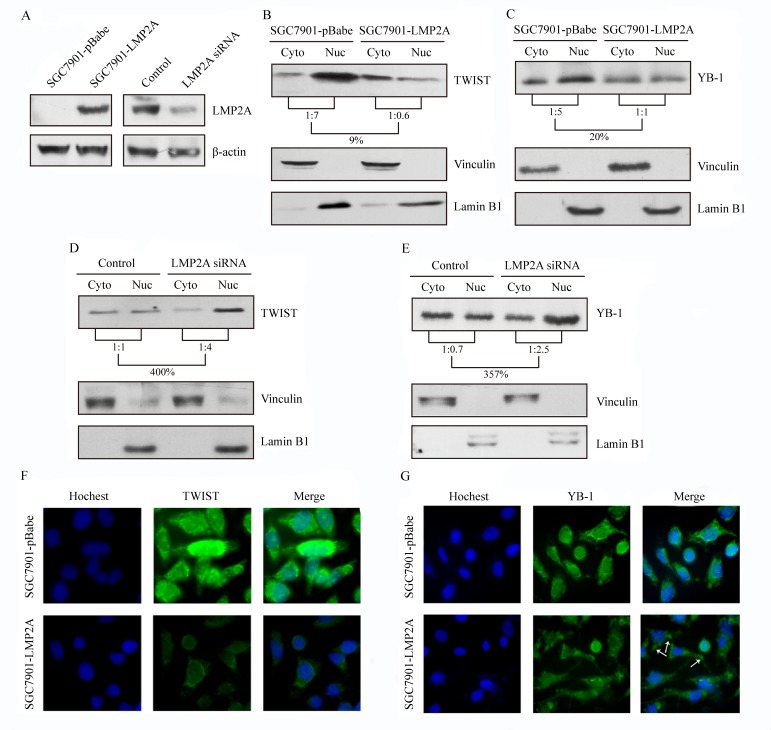
LMP2A down-regulated the compositional ratio of the nuclear fractions of TWIST and YB-1, and trigger their changes of subcellular localization (*A*) LMP2A was stably overexpressed in SGC7901 cells and transiently silenced in SNU719 cells for 48 h. Western blot analysis exibited the effect of the transfections. (*B, C*) The nucleus/cytoplasm ratio of TWIST (*B*) and YB-1 (*C*) decreased to 9% and 20%, respectively, after LMP2A was overexpressed in SGC7901-LMP2A cells. (*D*, *E*) Following LMP2A silenced in SNU719 siLMP2A cells, the compositional ratios of the nuclear fractions of TWIST (*D*) and YB-1 (*E*) were up-regulated to 400% and 357%, respectively. Vinculin and Lamin B1 were used as markers for the cytoplasmic and nuclear fractions respectively. (*F*, *G*) Immunofluorescent analysis showed that SGC7901-LMP2A cells have a substantial decrease in TWIST (green; *F*) and YB-1 (green; *G*) staining in both cytoplsmic and nuclear areas, and Hoechst staining was used to counter stain nuclei (blue).

As transcription factors, the nuclear protions of TWIST and YB-1 play the important role of triggering changes in HER2 expression [28-31]. To determine whether LMP2A is able to change the subcellular distribution of TWIST or YB-1, cytoplasmic and nuclear protein fractions prepared from SGC7901-LMP2A and SGC7901-pBabe cells were blotted and probed for TWIST, YB-1, vinculin and lamin B1. The nucleus/cytoplasm ratio of the TWIST and YB-1 proteins were decreased by 85% and 80%, respectively, in SGC7901-LMP2A cells relative to the vector-transfected cells (Figure 3B and 3C). Additionally, subcellular localization studies were performed on SNU719 cells that were transiently nucleofected with LMP2A siRNA or a universal siRNA control. The cytoplasmic and nuclear protein fractions were prepared 48 h after transfection. A 4.00-fold increase in TWIST (Figure 3D) and a 3.57-fold up-regulation in YB-1 (Figure 3E) were observed in the nuclear fraction of SNU719 siRNA cells when normalized to the cytoplasmic fraction.

Furthermore, SGC7901-LMP2A cells were plated on coverslips, fixed and stained for TWIST (green) or YB-1 (green) and nuclei (blue). Compared with SGC7901-pBabe cells, SGC7901-LMP2A cells exhibited a significantly decreased level of the TWIST (Figure 3F) and YB-1 (Figure 3G) proteins both in the nucleus and in the cytoplasm. There was a morphologic change of YB-1 to granular structures in the cytoplasm (white arrows) after LMP2A overexpression.

These results further showed that LMP2A can inhibit both the nuclear and cytoplasmic fractions of TWIST and YB-1, particularly the compositional ratio of the nuclear fraction.

### Effects of EBV, LMP2A and HER2 on overall survival

To evaluate the effects of EBV, LMP2A and HER2 on clinical outcome, overall survival data of our cohort were collected and 63 of 78 EBVaGC cases and all 216 EBVnGC cases had complete overall survival data.

**Figure 4:**
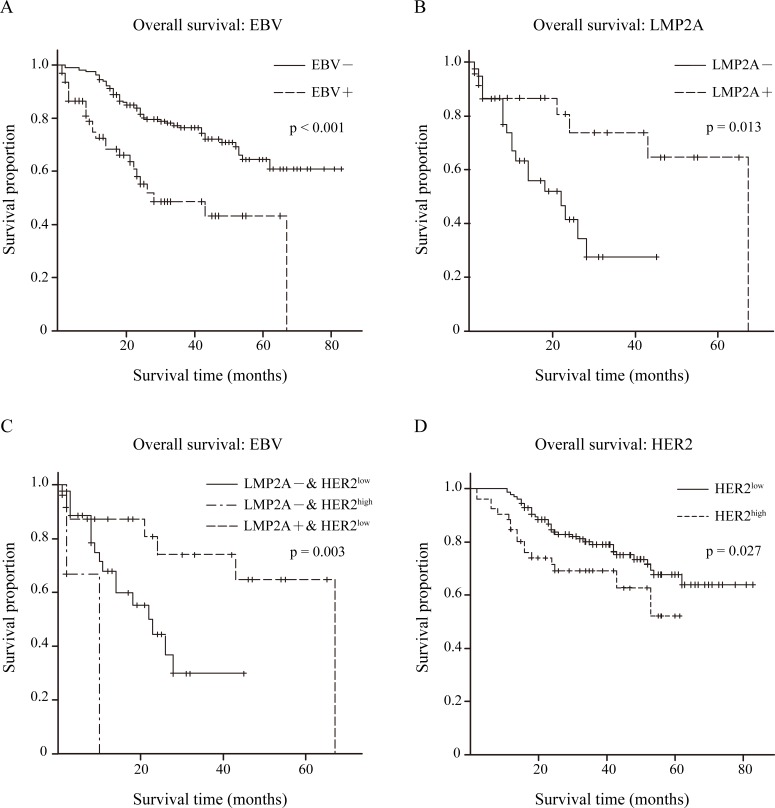
Overall survival condition within 279 GC patients (*A*) Kaplan-Meier curves for overall survival time in 279 GC patients according to EBV expression, p<0.001 by log-rank test. EBV– group n=216, EBV+ group n=63. (*B*) Kaplan-Meier curves for overall survival time in 63 EBVaGC patients according to LMP2A expression, p=0.013 by log-rank test. LMP2A– n=39, LMP2A+ n=24. (C) Kaplan-Meier curves for overall survival time in 63 EBVaGC patients based on both LMP2A and HER2 expression, p=0.003 by log-rank test. LMP2A– & HER2^low^ (0/1+) n=36, LMP2A– & HER2^high^ (2/3+) n=3, LMP2A+ & HER2^low^ (0/1+) n=24. (D) Kaplan-Meier curves for overall survival time in 216 EBVnGC patients according to HER2 expression, p=0.027 by log-rank test. HER2^low^ (0/1+) n=165, HER2^high^ (2/3+) n=51.

Initially, the effect of EBV on clinical outcome was analyzed. Kaplan-Meier overall survival curves were plotted, and a significantly longer overall survival was shown for EBVnGC compared with EBVaGC by log-rank tests (p<0.001; Figure 4A).

Of the 63 EBVaGC cases, the Kaplan-Meier overall survival curve of LMP2A− (n=39) and LMP2A+ (n=24) groups is depicted in Figure 4B, as well as the log-rank test result. Better overall survival was observed in the LMP2A+ group (p=0.013).

Next, we statistically analyzed the differences in overall survival time among the LMP2A−/HER2^low^ (n=36), LMP2A−/HER2^high^ (n=3) and LMP2A+/HER2^low^ (n=24) groups. The log-rank test showed that there was a significant difference among the three groups (p=0.003), and the LMP2A+/HER2^low^ group exhibited the best overall survival (Figure 4C).

In addition, overall survival data for the 216 EBVnGC cases were classified into HER2^low^ (n=165) and HER2^high^ (n=51) groups. The estimated product-limit survival functions of HER2 and the results of log-rank tests are shown in Figure 4D, and the HER2^low^ group exhibited better overall survival (p=0.027).

## DISCUSSION

In the present study, we found that HER2 expression is significantly reduced in EBVaGC compared with EBVnGC. Additionally, LMP2A, which is one of the EBV latency proteins, may participate in this reduction in HER2 expression in EBVaGC through a pathway involving TWIST and YB-1. Moreover, LMP2A+/HER2^low^ EBVaGC cases presented the best overall survival when compared with LMP2A−/HER2^low^ and LMP2A−/HER2^high^ EBVaGC cases.

In this cohort, 78 EBVaGC were identified from 1362 gastric carcinomas by EBER *in situ* hybridization, and the predominance of younger patient age, male gender and diffuse-type histology of EBVaGC is consistent with findings from recent studies [4,5]. Therefore, 216 EBVnGC cases, whose clinicopathological features were statistically matched with the 78 EBVaGC cases, were selected for further IHC study. This matching of clinicopathological features is helpful for eliminating the influence of clinicopathological features from revealing the relationship between EBV and HER2 expression in univariate analysis.

IHC analysis showed that HER2 expression is significantly reduced in EBVaGC compared with EBVnGC. In 2004, Lee HS *et al.* reported that 1 (1.6%) of 63 cases were high HER2 expressing in EBVaGC by IHC, which is less than in EBVnGC (13.5% of 281) [25]. Studies by Sukawa Y *et al.* in 2012 showed one case with HER2 overexpression of 18 EBVaGC cases (5.6%) compared with 19/213 (8.9%) EBVnGC cases [26]. Moreover, Bar-Sela G *et al.* demonstrated that none of 49 NPC cases, which is also an epithelial malignancy with consistent worldwide association with EBV, exhibited HER2 overexpression [32]. Consistent with these studies, this research suggests that EBV may contribute to down-regulation of HER2 expression in EBVaGC.

Because it belongs to the latency І pattern, EBVaGC expresses EBERs, BARTs and EBNA1. In addition, nearly half of EBVaGC express LMP2A [4,14]. In this study, 43.6% of EBVaGC cases showed LMP2A positive expression by IHC analysis. None of the 34 LMP2A-positive EBVaGC showed high HER2 expression, and all 4 HER2 high-expressing EBVaGC cases were LMP2A-negative. Statistical analysis showed no significant correlation between LMP2A and HER2 expression in EBVaGC. This non-correlation may be due to two factors: first, because of the low rate of high HER2 expression in EBVaGC, a larger EBVaGC cohort may be needed to reveal the statistically significant relationship between LMP2A and HER2 expression; second, there are other fragments encoded by EBV that may suppress HER2 expression in EBVaGC simultaneously, such as EBNA1 which was reported to be a suppressor of HER2 in human ovarian cancer [33,34]. However, ovarian cancers, in which EBV is not presented based on EBER *in situ* hybridization, are not actually EBV-associated epithelial malignancies [4]. Whether EBNA1 could also suppress HER2 expression in EBVaGC remains unknown and needs to be further studied.

Karlra J *et al*. suggested that the TWIST-YB-1 axis was involved in the regulation of HER2 expression [29]. TWIST, a basic helix-loop-helix transcription factor, is known to bind to E-box regions within the YB-1 promoter and thus regulate YB-1 expression [35,36]. Additionally, TWIST is reported to be expressed in gastric cancer, breast cancer, hepatocellular carcinoma, prostate cancer, esophageal squamous cell carcinoma, bladder cancer and pancreatic cancer and its nuclear localization contributes to the up-regulation of YB-1 expression [37,38]. YB-1 was initially reported to interact with the Y-box element in the 5′ regulatory element of the HER2 gene [39], and nuclear YB-1 can trigger HER2 expression, which was confirmed by CHIP assay in gastric carcinoma cell lines MKN45, SNU216, and NCI-N87 [28,31,40].

In this study, overexpressing LMP2A can down-regulate expression of TWIST, YB-1 and HER2 in the EBV-negative cell line SGC7901, siRNA silencing of LMP2A can up-regulate TWIST, YB-1 and HER2 expression in the EBV-positive cell line SNU719, and LMP2A overexpression can decrease both nuclear and cytoplasmic fractions of TWIST and YB-1, especially the compositional ratio of the nuclear fractions.

Interestingly, using immunofluorescent analysis to locate YB-1 in SGC7901 cells, some granular structures formed by YB-1 were observed in the cytoplasm following LMP2A overexpression. J Kalra *et al.* showed the same morphologic change in YB-1 when YB-1 was suppressed in breast cancer cell lines LCC6^Her2^ and SKBR3 [29]. These granular structures may be stress granules (SGs) which are thought to be sites of stalled translation and appear when YB-1 is suppressed. By formatting these SGs, progress of HER2 translation is inhibited [41-43]. Hence, tests *in vitro* suggested that LMP2A could down-regulate expression of HER2 in gastric cell lines, and this reduction in HER2 expression induced by LMP2A may be achieved through the TWIST-YB-1 axis.

To further evaluate the effect of inhibition of HER2 expression via LMP2A on prognosis, we analyzed overall survival data for 63 EBVaGC that were sub-grouped according to LMP2A and HER2 expression. LMP2A+/HER2^low^ EBVaGC cases had better overall survival compared with LMP2A−/HER2^low^ and LMP2A−/HER2^high^ EBVaGC cases, and the last group exhibited the worse overall survival.

Meanwhile, we explored a possible correlation between LMP2A expression and the overall survival of EBVaGC. We showed that patients with LMP2A expression had a better overall survival rate than those without LMP2A expression in EBVaGC. Thus far, no dedicated article has presented a correlation between LMP2A expression and prognosis in EBV-associated epithelial malignancies, including NPC and gastric carcinoma. While Mao *et al.* reported that LMP2A-positive cases had a significantly lower overall survival rate than LMP2A-negative cases in nasal type NK/T lymphomas [44]. This inconsistency may be due to the difference in tumor type and latency type (NK/T lymphoma is latency II neoplasm) [4].

In addition, worse overall survival was observed in EBVaGC compared with EBVnGC, but pooled analysis from Camargo MC *et al.* presented a significantly better survival time for EBVaGC than EBVnGC [10,11]. Van Beek *et al.* reported that no difference was observed in overall survival of EBVaGC (n=41) compared with EBVnGC (n=525) [13]. We think there are three possible reasons for these results. First, the clinicopathological features may influence the clinical outcome in gastric carcinoma. For example, advanced tumor stage, lymph node metastasis and older age were correlated with poor prognosis [45]. The EBVaGC and EBVnGC cases in the above studies were not always clinicopathological-feature matched. In the present study, however, we used clinicopathological-feature matched EBVaGC and EBVnGC cases, which may eliminate the influence of clinicopathological features on prognosis when univariate analysis was performed. Second, in the pooled analysis from Camargo MC *et al.,* some studies defined EBVaGC by qRT-PCR testing and not EBER *in situ* hybridization. The qRT-PCR testing cannot avoid the interference of lymphocytes in the stroma, which can also be infected with EBV. Thus, identifying EBVaGC by qRT-PCR does not conform to the definition of EBVaGC, which is that EBV must be proved to be present in gastric carcinoma cells by EBER *in situ* hybridization [10,11,13]. Last, the inconsistency among these studies may also be due to geographic location and ethnic variations.

In conclusion, this study indicates for the first time that there is reduced expression of HER2 in EBVaGC compared with EBVnGC based on a clinicopathological-feature matched cohort. This reduction may be induced by LMP2A through the TWIST/YB-1 axis in EBVaGC. Moreover, LMP2A+/HER2^low^ EBVaGC cases may be correlated with a better prognosis than LMP2A−/HER2^low^ and LMP2A−/HER2^high^ EBVaGC cases. These findings extend our knowledge about EBV such that although EBV is recognized as an oncogenic virus, some products of EBV, such as LMP2A, may down-regulate HER2 expression and possibly be helpful to improve outcomes in gastric carcinoma patients. Further studies are needed to precisely confirm the functional domain of LMP2A, which can down-regulate HER2, avoid LMP2A's own oncogenic potential, and serve as a potential target for treating gastric carcinoma with high HER2 expression.

## MATERIALS AND METHODS

### Ethics statement

The use of human subjects was approved by the Clinical Research Ethics Committee of the Third Affiliated Hospital, Sun Yat-sen University, Guangzhou, China. All adult subjects provided informed consent, and a patient or guradian of any children participant provided informed consent on their behalf. Ethical guidelines under the Declaration of Helsinki were followed.

### Tumor tissue microarrays

The files of 1362 surgical resection gastric adenocarcinoma cases were collected at the First, Second and Third Affiliated Hospitals of Sun Yat-sen University and the Guangzhou First Municipal People's Hospital, Guangzhou, southern China from January 1, 2000 to December 31, 2012.

Twenty-nine tissue microarray (TMA) blocks, containing the total of 1362 cases, were conducted as described by Chen JN *et al* [8]. Four μm thick sections were cut from each tissue array block to further detect EBV infection status using an *in situ* hybridization assay for EBV-encoded small RNA-1 (EBER-1). EBER-1 (+) and EBER-1 (−) cases were defined as EBVaGC and EBVnGC, respectively. Of the 1362 cases, 78 cases (5.7%) were identified as EBVaGC.

### *In situ* hybridization (ISH)

An ISH assay was performed on the TMA block sections with an EBV oligonucleotide probe complementary to the EBER-1 (PanPath, Amsterdam, Netherlands), according to the manufacturer's instructions. Sections from a known EBER-1-positive NPC tissue were used as the positive controls and a sense probe for EBER-1 was used as the negative control.

### Tissue specimens

Clinical-pathological parameter-matched 78 EBVaGC and 216 EBVnGC were selected from the 1362 GC cases. Clinicopathologic data were obtained from the archives of the four Departments of Pathology. Histology of the gastric carcinomas was classified as intestinal- and diffuse-type, according to the Lauren classification [46]. Cancer staging was classified according to the TNM cancer staging system of the American Joint Committee of Cancer [47]. The clinical outcome was followed up from the date of gastric carcinoma resection until the date of death from patients directly or families by phone-call or letters. The follow-up period was 1-83 months (median 26 months)

### Immunohistochemical staining

The Envision Immunohistochemistry (IHC) system was used to analyze the expression of HER2 and LMP2A [48]. The slides were incubated with a rabbit-derived polyclonal antibody against HER2 (A0485, 1:200 dilution; Dako, Copenhagen, Denmark) or a mouse-derived monoclonal antibody against LMP2A (clone 4A11B3A3, 1:100; a gift from Prof. Mu-sheng Zeng) as the primary antibody [49] and a HRP-labeled secondary antibody (Dako Envision). A HER2-positive breast carcinoma and a LMP2A-positive NPC case were used as positive controls and substitution of the primary antibodies with TBS was used as a negative control.

The HER2 IHC staining results were scored into 0, 1+, 2+ and 3+ according to the criteria recommended by the ToGA test [23]. And LMP2A was localized on the cell membrane and in the cytoplasm of the tumor cells. All of the slides were evaluated by two experienced pathologists without knowledge of the patient or patients' clinical status.

### Cell lines and transfections

The EBV-negative SGC-7901 human gastric carcinoma cell line (Cell Bank of Type Culture Collection of Chinese Academy of Sciences, Shanghai, China) and the EBV-positive SNU-719 human gastric carcinoma cell line (Korean Cell Line Bank, Seoul, Korea) were maintained in Roswell Park Memorial Institute (RPMI) 1640 medium (Gibco) supplemented with 10% fetal bovine serum (FBS) in a humidified 5% CO_2_ incubator at 37°C. All of the cell lines were tested to ensure that they were mycoplasma free.

Recombinant retroviruses expressing either vector pBabe or pBabe subcloned with LMP2A were gifted from Dr. Mu-Sheng Zeng (Sun Yat-Sen University Cancer Center, Guangzhou, China) [49] and the plasmid DNA was transfected into SGC-7901 cell lines by using Lipofectamine 2000 (Invitrogen, Carlsbad, CA, USA) according to the manufacturer's instructions. Pooled SGC-7901 cell populations expressing either pBabe or pBabe-LMP2A were selected with 0.5 μg/ml of puromycin (Sigma-Aldrich, St. Louis, MO).

### Small RNA interference

Target siRNA duplex and control scrambled siRNA duplex were synthesized by Invitrogen (Carlsbad, CA, USA). The siRNA target sequence to LMP2A mRNA is 5′-AACUCCCAAUAUCCAUCUGCU-3′, which does not overlap with the sequences of LMP2B [50]. The siRNA duplex was transfected using Lipofectamine RNAiMAX Reagent (Invitrogen) as recommended by the manufacturer, and the cells were assayed for silencing one or two days after transfection.

### RNA extraction and real time RT-PCR

Total RNA from different cell lines was extracted by using the E.Z.N.A. Total RNA Kit I (OMEGA, USA). The purity of each RNA sample was determined by assessing the A260/A280 ratio, which consistently measured between 1.6 and 1.8. The RNA was reverse transcribed using the PrimeScript 1st Strand cDNA Synthesis Kit (TAKARA, Dalian, China) according to the manufacturer's instructions. Then, real-time quantitative reverse transcription-polymerase chain reaction (RT-PCR) analysis was performed on an ABI 7500 FAST Real-Time PCR System by using SYBR^®^ *Premix Ex Taq*™ II (TAKARA). Quantitative determination of RNA levels were performed in three independent experiments. The housekeeping gene GAPDH was used as an internal control to normalize the expression levels of different genes. The primers used were listed in Supplementary Table S1.

### Western blot analysis

Western blotting analysis was used to semiquantitatively determine LMP2A, TWIST, YB-1 and HER2 protein levels. The primary antibodies used were anti-LMP2A (a gift from Prof. Mu-sheng Zeng), anti-TWIST (H81; Santa Cruz Biotechnology, Santa Cruz, CA, USA), anti-YB-1 (D299), anti-HER2 (D8F12), anti-β-actin (13E5), anti-vinculin (E1E9V) and anti-Lamin B1 (D9V6H; Cell Signaling Technology, Beverly, MA, USA).

For whole-cell proteins studies, proteins were harvested by homogenizing cells in lysis buffer (50 mM Tris/HCl pH 8.5, 150 mM NaCl, 0.02% sodium azide, 0.1% SDS, 1% NP-40 and 0.5% sodium deoxycholate). Protein concentrations were determined using the Bio-Rad protein assay (Bio-Rad, Richmond, CA). Equal amounts of samples were separated on 8% or 10% SDS-PAGE gels, and transferred to polyvinylidene fluoride membranes (Millipore, Bedford, MA, USA). The blots were then probed with the appropriate HRP conjugated secondary antibodies and visualized using ECL (Thermo). The experiments were conducted at least three times. The results were quantified using Quantity One v4.62.

For subcellular localization studies, nuclear and cytoplasmic proteins were separated with NE-PER Nuclear and Cytoplasmic Extraction Reagents (Thermo, Rockford, IL, USA) after whole-cell proteins extracted. Other steps of immunoblot were the same as whole-cell proteins studies.

### Immunofluorescence analysis (IF)

Cells grown on coverslips were fixed using a 4% paraformaldehyde solution in phosphate-buffered saline (PBS), permeabilized with 2.5% Triton X-100 at room temperature before staining with primary antibody of YB-1 or TWIST, the same antibodies used for western blotting, at a dilution of 1:100 or 1:1, respectively. Primary antibody binding was detected by further incubations with anti-rabbit or anti-mouse Alexa488 (Cell Signaling Technology), respectively. Hoechst (Molecular Probes, Eugene, OR, USA; 1:1000) was used to visualize the nuclei. The cells were viewed using a Leica fluorescent microscope. Images were captured using DC100 digital camera and Open Lab software.

### Statistical analysis

A chi-square test, Fisher's exact test or Kruskal Wallis test was used for statistical analysis of the IHC results and clinicopathologic findings. And the expression of mRNA or proteins was analyzed using non-paired Student's t-test. Overall survival analyses were performed using the Kaplan-Meier method and log-rank test. The overall survival time to event was defined as the time of surgery until death, irrespective of the cause of death. The results were considered to be statistically significant at a p-value of less than 0.05. All of the p-values presented in this study are two-sided. All of the statistical analyses were performed with the SPSS 13.0 statistical software program for Windows (SPSS Inc., Chicago, Illinois, USA).

## SUPPLEMENTARY MATERIAL TABLE



## Abbreviations

HER2: human epidermal growth factor receptor-2
EBV: Epstein-Barr virus
EBVaGC: EBV-associated gastric carcinoma
EBVnGC: EBV-negative gastric carcinoma
LMP2A: latent membrane protein 2A
EBNA: EBV nuclear antigen
EBER: EBV-encoded small non-coding RNA
BARTs: BamHI A rightward transcripts
