# Supplementary material for: Epstein-Barr virus latent membrane protein 2A suppresses the expression of HER2 via a pathway involving TWIST and YB-1 in Epstein-Barr virus-associated gastric carcinomas
Source: Oncotarget. 2014 Nov 6;6(1):207–20. doi: 10.18632/oncotarget.2702 (PMC4381589; doi:10.18632/oncotarget.2702)
Supplement: Supplementary file 1 [file oncotarget-06-207-s001.pdf]

Epstein-Barr virus latent membrane protein 2A suppresses the expression of HER2 via a pathway involving TWIST and YB-1 in Epstein-Barr virus-associated gastric carcinomas

Supplementary Material

Supplementary Table S1: List of primers used in the present study.

Primers*	Sequence (5' to 3')
**HER2**	
Sense	GCCTGTGCCCACTATAAGGAC
Antisense	GCAGCTTCCGCATCGTGTA
**LMP2A**	
Sense	ACGATGGCGGAAACAACTC
Antisense	GGGTCCTCATAAGGCGGTG
**RT-siRNA-LMP2A**	
Sense	GAATTCTGCAGCTATGGGGTCCCTA
Antisense	AGATCTGCGATCTGGTGGGCATTC
**YB-1**	
Sense	CGCAGTGTAGGAGATGGAGAG
Antisense	GAACACCACCAGGACCTGTAA
**TWIST**	
Sense	GGAGTCCGCAGTCTTACGAG
Antisense	CCAGCTTGAGGGTCTGAATC
**GAPDH**	
Sense	CCACCATGGAGAAGGCTGGGGCTCA
Antisense	ATCACGCCACAGTTTCCCGGAGGGG

*The amplification protocol for these five regions was one cycle at 94°C for 5 min, followed by 40 cycles of denaturation at 94°C for 1 min, annealing at 57°C for 1 min and elongation at 72°C for 90 sec, and a final extension at 72°C for 10 min.
